# Graph Machine Learning Can Estimate Drug Concentrations in Whole Blood from Forensic Screening Results

**DOI:** 10.1021/acs.analchem.5c07428

**Published:** 2026-03-18

**Authors:** Tetiana Lutchyn, Marie Mardal, Michael Nedahl, Benjamin Ricaud

**Affiliations:** † Department of Physics and Technology, 8016 The Arctic University of Norway, 9019 Tromsø, Norway; ‡ Department of Pharmacy, 8016 The Arctic University of Norway, 9019 Tromsø, Norway; § Department of Forensic Medicine, 4321 University of Copenhagen, 2100 Copenhagen, Denmark

## Abstract

LC-HRMS is widely used in forensic toxicology for broad-scope screening. When a newly emerging or rarely encountered compound is tentatively identified, toxicologists must decide whether it may be relevant to a case and, if so, quantify it. However, acquiring reference material for quantification is costly and time-consuming. A rapid semiquantitative estimation method would help prioritize only compounds above the toxic threshold. This study presents a machine-learning (ML) framework that estimates drug concentrations in whole blood using molecular structure information and LC-HRMS signals. Using a data set of 191 drugs spiked into whole blood at multiple concentration levels, we trained and evaluated several ML models. Standard models, including Random Forests, achieved moderate performance. In contrast, a recently reported Graph Neural Network (GNN) leveraging atomic features and global molecular properties consistently produced the highest accuracy. Under cross-validation, the GNN predicted signal-to-concentration ratios for 79% of all molecules, corresponding to concentration estimates between 50% and 200% of the true value. Toxicological thresholds often span multiple orders of magnitude, making this precision acceptable. The GNN model was additionally evaluated on an external benchmark data set of ionization efficiencies (*logIE*), where it outperformed the current state of the art. Overall, the results demonstrate the feasibility of using graph-based ML to estimate drug concentrations in whole blood without reference material. This is a practical ML tool that can support decision-making in toxicological evaluation, particularly for newly emerging or rarely encountered drugs. The GNN model is open source, and the data set used for training and testing the models are publicly available.

## Graphical abstract

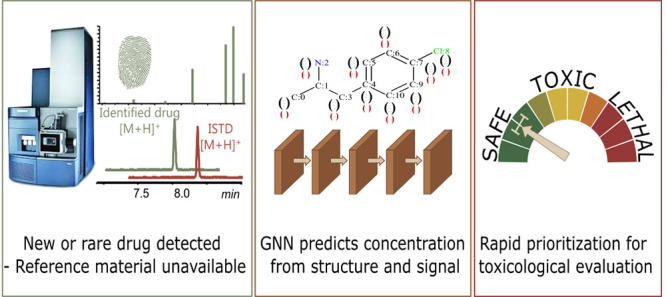

## Introduction

Liquid Chromatography–High Resolution Mass Spectrometry (LC-HRMS) is widely used in forensic toxicology for broad-scope screening as an integrated part of the systematic toxicological analysis. Uncommon or novel drugs are occasionally detected in blood samples during forensic investigations. These may include drugs not marketed in the laboratory’s country, pesticides, or new psychoactive substances. In some cases, these drugs can be reliably identified using suspect screening workflows.^1,2^ If the identified drug could be relevant for the forensic case, then the forensic toxicologist may decide to acquire or synthesize a reference standard for confirmation and quantification. However, since the toxicological evaluation depends on the measured concentrations, it is difficult to decide whether the drug is relevant without a quantitative estimate.

A computational model capable of estimating the concentration of an identified drug in a blood sample can in these cases serve as automated decision-support. It can help prioritize cases involving potentially toxic concentrations and reduce workload by excluding substances present at subtoxic levels.

Machine learning approaches have become increasingly integrated into analytical chemistry workflows, supporting tasks such as signal processing, compound identification, and quantitative prediction.^3,4^ Within the field of forensic toxicology, machine learning applications have particularly focused on the identification of new psychoactive substances and retention time predictions.^5–7^ Research on quantification without reference material with ML is rapidly evolving, particularly for the prioritization of environmental pollutants.^8–10^ The prediction of concentration from LC-HRMS signals has mainly focused on the prediction of ionization efficiency (IE), which is transferable between analytical methods.

The quantification of drugs in whole blood samples is commonly performed using the interpolation of signals measured in unknowns with calibration curves based on spiked standards in blind matrices. The sensitivity, reproducibility, and correlation between signal and concentration depend on the drug, instrument, and method. Internal standards (ISTDs) are used to correct for technical variation. In a recent interlaboratory comparison of LC-HRMS quantitation approaches, the use of predicted IEs was found to be superior to the use of surrogate standards; 83% of the evaluated compounds were predicted within 1 order of magnitude of the correct concentration across different instrumentation and laboratories.^9^ This approach assumes that the molecule is in solution and that there is no matrix effect. However, for a concentration prediction model to be of value in high-throughput toxicological screening, it should predict the concentration of a given drug in the matrix, with the sample preparation method and analytical method used for standard toxicological analysis. For this reason, we develop an ML model trained on data from matrix-matched spiked reference material.

We here propose an approach that predicts the signal-to-concentration ratio from the structure of the molecule. A recently published graph machine learning (ML) method—developed and validated for predicting molecular properties of small organic molecules—serves as the basis for the analysis.^11^ The approach presupposes accurate identification of suspects and signals with good peak shape (i.e., chromatographically retained, not splitting, and not overloaded). The overall process is depicted in Figure 1. From a sample where a molecule *m* is at concentration *c*_*m*_ in whole blood, LC-HRMS gives a signal area denoted *s*_*m*_. We introduce *s̅*_*m*_ = *s*_*m*_/*s*_ISTD_ ISTD-corrected area (for more detail, see the section on data acquisitions) and the signal-concentration ratio α_*m*_ of the molecule *m*:

$$\alpha_{m}=\frac{{\mathrm{s̅}}_{m}}{c_{m}}$$ 1

In this work, we propose to estimate the parameter α_*m*_ using exclusively information about the molecule *m*, its structure, and properties, independent of its concentration in the sample. We show that our ML methods can indeed deduce α_*m*_ from the structure of the molecule, providing a useful connection between the area and the concentration of the molecule in a sample.

**Figure 1:**
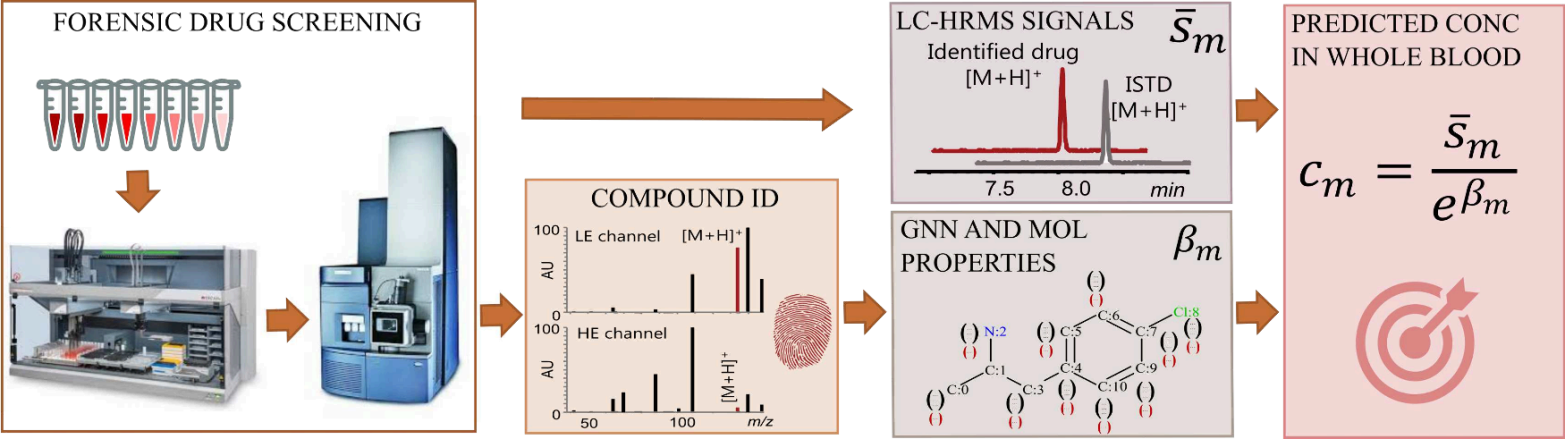
Spiked drugs were analyzed at different concentration levels using a forensic drug screening method. Molecular properties and graphs were derived from the analyzed drugs. From this input, the GNN model is developed to estimate drug concentrations in whole blood.

We make use of different ML models: Multi-Layer Perceptron, Decision Tree, and Random Forest, as well as a more advanced model based on a Graph Neural Network (GNN). We show that first it is possible to predict α_*m*_ with high accuracy using ML methods, in particular with Random Forest, and second that the best model is the GNN which outperforms all the other approaches.

Since the ratio α_*m*_ can take values that differ by several orders of magnitude (3 for our data set), we apply the natural logarithm to α_*m*_, and focus on evaluating this logarithmically transformed value instead:

$$\beta_{m}=\ln\left(\alpha_{m}\right)$$ 2

Reducing the variability of the quantity to learn within 1 order of magnitude is helpful for ML methods. We will then focus on predicting the quantity β_*m*_ throughout this manuscript.

The present study is the first attempt to estimate the concentration of drugs in whole blood from molecular properties and LC-HRMS signals without the use of reference standards. The aim of this study was to use a recently published GNN model, developed for the prediction of molecular properties of small organic molecules, and use it to predict drug concentration levels in whole blood from a previously acquired data set. The GNN model used here has been introduced in previous work^11^ as a lightweight but efficient model for predicting molecular properties. It is a hybrid method that combines a common GNN approach, in which molecules are seen as graphs of atoms, together with additional chemistry knowledge. To benchmark our findings, the produced models are applied to a second data set with electrospray IEs used in Aalizadeh et al.,^10^ which is related to the task of predicting β.

## Materials and Methods

### Materials and Reagents

LC–MS grade methanol, water, formic acid, and acetonitrile were obtained from Fisher Scientific (Loughborough, UK). Chemicals of analytical grade or higher and reference standards were obtained from different suppliers or pharmaceutical companies. Blank pooled human whole blood, preserved with 1% sodium fluoride, was obtained from blood donors (Blood Bank, Rigshospitalet, Copenhagen, Denmark).

### Data Acquisitions

Spiked whole blood was prepared at multiple concentration levels, to evaluate the screening method for decision points of common drug targets. The decision point is the administratively defined cutoff or concentration that is at or above the methods limit of detection or limit of quantitation and is used to discriminate between positive and negative results. These studies are resource-intensive - but recommended in ANSI/ABS Standard 036 (Standard Practices for Method Validation in Forensic Toxicology) to evaluate screening performance and interpret results. The results are used in routine forensic toxicological investigations and are reused for this study to evaluate if it is possible to also estimate the concentration of drugs without having to perform these experiments. A detailed description of the data acquisition is available in the Supporting Information.

Data acquisition: Dilution rows at seven concentration levels of 13 different standard mixtures were prepared by serial dilution. Each standard mixture had 7–35 different drug targets (median: 20) with a total of 231 drug targets. The diluted mixtures were spiked to blank whole blood samples with a concentration range of typically 0.001–0.1 mg/kg. These spiked whole blood samples were then extracted by protein precipitation using a Tecan Freedom EVO 200 robotic platform (Tecan Group Ltd., Männedorf, Switzerland).^12^ Analyses were performed using an Ultra-High Performance Liquid Chromatography - quadrupole Time-of-Flight - Mass Spectrometry system consisting of an ACQUITY UPLC I-Class coupled to a Xevo G2-S QTOF (Waters, Milford, MA, USA). Analytes were separated using an ACQUITY UPLCHSS C18 (1.8 μm 2.1 × 150 mm) column (Waters). The mass spectrometer was operated in positive electrospray ionization mode, and data were acquired using a data-independent acquisition mode with elevated collision energy. The instrument was operated with UNIFI 1.9 (Waters). The samples were prepared and analyzed on at least two different runs. Quality control of analytical runs consisted of system suitability testing, evaluation of solvent blank samples, and evaluation of known drugs in matrix-matched QCs.

The data were extracted from a database comprising unannotated LC-HRMS screening data. As part of the preprocessing in the vendor software, the peaks had been centroided in the mass and retention time domains and were then parsed to an SQL database, as previously described.^13^ For this study, data from the selected experiments were extracted as an area of [M + H]^+^ from the low-energy channel spectra. Information from the high-energy channel was not used. Using samples names as ID, counts (a vendor intensity measure) for drugs and ISTD (mianserin-D_3_) were extracted with ± 0.25 min and ± 0.001 *m*/*z* from library values. Only values from 0.7 to 12.5 min were considered.

### Preprocessing of Analytical Data

The extracted data contain a set of 3,653 concentration-drug combinations. The analytical measurements contain some technical noise, rounding of concentration values, and other external factors. For each molecule *m*, α_*m*_ is computed for all measurements and averaged to mitigate the noise. This average is denoted ${\mathrm{\alpha ̅}}_{m}$ . In Figure 2, we provide the distribution of the values around the average for all measurements. Fifteen measurements among the 3,653 were defined as outliers from the linear range of the signal-to-concentration curve, as they had values with a deviation of more than ± 0.5*α̅*_*m*_ from the mean. These values were removed from the data set.

**Figure 2:**
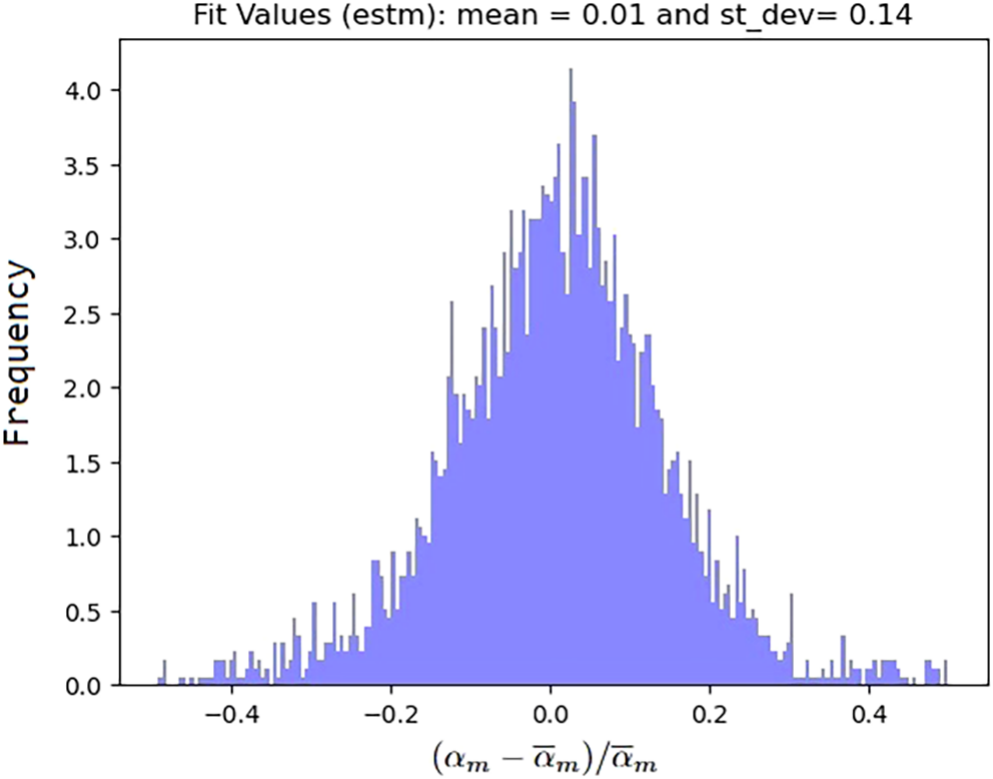
Distribution of measured values for α_*m*_ around their mean value ${\mathrm{\alpha ̅}}_{m}$ for all molecules. The horizontal axis is $\left(\alpha_{m}-{\mathrm{\alpha ̅}}_{m}\right)/{\mathrm{\alpha ̅}}_{m}$ , and the vertical axis gives the density.

We then focus on predicting ${\mathrm{\alpha ̅}}_{m}$ , as one value per molecule. For the sake of simplicity, unless otherwise stated, we drop the bar and use α_*m*_ for the mean value henceforth. These mean α_*m*_ values are in the range of [0.795, 1080] for the 208 molecules.

As a further preprocessing step, 58 values of the samples (2% of the data) were removed due to unreasonable values. In addition, caffeine, phenobarbital, 19-norandrosterone and 2-hydroxymethylolanzapine were removed from the data set due to the high levels in blank samples or matrix interference. After this filtering, the final data set consisted of 191 molecules.

### Benchmark Data Set: Prediction of Ionization Efficiency

We used a second data set to test our models. This data set is openly accessible and was first analyzed in.^10^ It contains IE values for a library of 103 emerging contaminants, expressed on a logarithmic scale referred to as *logIE*, determined by LC-HRMS. For 11 of the molecules in this data set, it was not possible to find their SMILES specification in PubChem. As a consequence, we excluded them from our tests and included only 92 molecules.

### Data Preprocessing for Machine Learning

Information about the structure of the molecule is crucial for our prediction task. To collect it, we use the SMILES encoding and publicly available resources. We have two different families of ML models that require two different sets of input data. The first one requires a list of features for each molecule. This will be a vector of real values (the same size for all molecules) containing the different molecular properties that we have collected. The second family is that of GNNs, which focus on processing the molecule as a graph. For the latter method, we need to give as input the graph structure of a molecule as well as the properties of the atoms (nodes of the graph) that it contains.

#### Molecular Fingerprints for Machine-Learning Models (Except GNNs)

To collect the molecular fingerprint or properties of our molecules, we use the open-source library ”Padel”,^14^ described by Yap.^15^ Since we do not know apriori what properties are important for our task, we collect as many of them as possible. We have collected 1444 molecular features per molecule, including information about chemical elements, their structure, and their spatial shape. In a second step, we drastically reduce the number of these molecular properties to 11 by leveraging the estimation of feature importance provided by the Random Forest approach. Indeed, important features can be defined as the one that decreases the most impurity during splitting at the tree level of the Random Forest.^16^

#### Graphical Representation for the GNN Model

For the GNN, each molecule is seen as a graph where atoms are nodes, and bonds between atoms are graph edges. The RDKIT Python toolbox^17^ provides the molecular graphs and atomic features from the SMILES encoding. For each molecule, the input data are made up of two mathematical objects. The first is a graph of the bonds between atoms (adjacency matrix or list of edges). The second is a matrix containing information about the atoms in the molecule: Each row corresponds to an atom, and each column is an atomic descriptor or ”feature”. The selected atomic features for the proposed GNN model are as follows: atomic number, number of hydrogen atoms, number of valence electrons, number of radical electrons, formal charge, type of hybridization, degree of atom, whether the atom is part of a ring or an aromatic ring in the molecule, and scaled versions of: the van der Waals radius, atomic mass, and covalent radius.

In addition to these atomic descriptors, we include on the nodes of the graph some information derived from the global structure of the molecule. These global property values are copied directly on all atoms of the molecule, as in Lutchyn et al.^11^ This helps the GNN to access important molecular global properties more easily. We add the volume, width, length, and height of the molecule in 3D provided by RDKIT.

As in Lutchyn et al.,^11^ in the graph we do not encode the type of bonds between atoms in the molecule, only whether or not two atoms are bound.

### Machine Learning Modeling and Validation

Owing to the small size of our data set to predict β (191 molecules), we perform an extensive validation approach. We use two types of cross-validation (CV): Leave One Out CV (LOOCV) and 5-fold CV. For the 5-fold CV, we used nonstratified random splitting, given the relatively small data set and continuous regression targets; however, target distributions were checked to confirm reasonable balance across folds. We used a fixed random seed of 42 for reproducibility and report the results of a single run. In addition, we perform LOOCV on different subsets (of randomly selected samples) of the data set, of varying sizes. This enables a more robust and in-depth validation of the results and an evaluation of the effect of the data set size on the models performance. For the *logIE* data set (92 molecules), which is smaller and is only used as a benchmark, we use LOOCV alone. We use the following ML methods.

#### Multi-Layer Perceptron (MLP) Regression

We use an open-source version of MLP available as part of the Scikit-learn Python library.^18^ We designed several architectures using a grid search approach and our best model is a 5 hidden layers network with number of nodes (500, 400, 300, 200, 100), nonlinear Relu activations between layers, and mean squared error (MSE) loss, and trained using the Adam solver. MLP regression models with 3 and 4 hidden layers were also evaluated with various embedding sizes and nonlinear function, following a grid search approach. Input features were standardized prior to training. No significant improvement in predictive performance was observed across the different network architectures tested. The results were all bad for the training on all features and better, but not significantly different for the training on 11 features.

#### Decision Tree Regression

We use the open-source Scikit-learn version,^18^ with the default parameters (mean square as criterion, min sample split of 2) and trained with a maximum depth of 4. Depths of 3 and 5 where tested with worse results. Different Decision Tree models were evaluated using MSE-based splitting criteria, minimum split sizes of 2 to 5, minimum leaf sizes of 1–2, and both squared error and Friedman MSE criteria. Varying these parameters did not lead to performance gains.

#### Random Forest Regression

The main parameters for training the Random Forest Regression^18^ are a maximum depth of 4 for the trees and a number of estimators of 500. Random Forest models were evaluated over a range of two additional depths (3 and 5), number of estimators (200 to 800 with step 100), and different node-splitting parameters.

#### Graph Neural Network Model

We use the model introduced in our previous work.^11^ It is a relatively small GNN with Graph Attention (GAT) layers.^19^ This model was made from the open-source Pytorch Geometric library. The input data for this model are the molecular graph as well as 16 elemental properties that are the node features *h*. We add 4 global features to the 12 local features for each node to represent the general shape of the molecule: its volume, width, length, and height. The model is configured with four GAT layers for our library (β values) and five layers for the *logIE* library, with the activation function ‘tanh’. The optimizer is Root Mean Squared Propagation (RMSprop). The number of channels (embedding size) for each hidden layer is set to 28. The best hyperparameters were found using a grid search.

In the output of the GNN, we obtain one value per node. However, we want to predict one value per molecule. This is generally done by adding a pooling layer as the last layer of the GNN. It either averages the values over the nodes or takes the maximum or the minimum of the values. Nevertheless, this may not be optimal.^11^ The hypothesis is that the predicted value may not depend on the full molecular structure but only on a substructure. Therefore, global pooling may be less efficient. However, we will see in the results that it does not make much difference compared with the commonly used pooling approach for our data sets.

The GNN architecture was chosen to balance model complexity and the limited size of our data sets. The activation function tanh was selected for its ability to model both positive and negative interactions in the node embeddings, which is relevant for capturing subtle variations in chemical properties. The embedding size of 28 channels per hidden layer was determined through a grid search that optimized validation performance while avoiding overfitting; larger embeddings tended to overfit given the small data set (191 molecules). Similarly, the number of layers was chosen to allow sufficient message passing across the molecular graph without causing oversmoothing, which can occur with deeper GNNs. The feature sets were restricted to atomic and bond information (and selected global molecular descriptors in the hybrid approach) because these provide the most chemically meaningful information while keeping the input dimensionality manageable for small data sets. Overall, the architecture and hyperparameters reflect a trade-off between expressive power, training stability, and generalization to limited data, ensuring that the GNN captures relevant molecular information without overfitting.

## Results

The predictive performance of various ML models and the GNN was evaluated using cross-validation and an external ionization-efficiency benchmark data set. Since we perform a regression task (predicting the value of β across molecules), we report the MSE. However, we noted several outliers in the data (i.e., predictions with a large deviation from the ground truth). Outliers have also been reported in previous work on predicting IE^9^ and concentration errors appear to stem from inaccuracies in response factor prediction rather than from concentrations falling outside linear range.^20^ The MSE score is highly sensitive to outliers and can give a false impression of low quality regression. Therefore, we provide additional measures for evaluating our models, which are more robust to outliers. Specifically, we use the mean average error (MAE) and introduce a second, simpler and more intuitive measure, related to our application and experimental setting: a classification score. We will call this measure *accuracy* and denote it by *A*. The idea is to introduce a threshold *t* and count a prediction as correct if it falls within some defined interval, ± *t* around the correct value. *A* is defined as follows:

$$A=\frac{1}{N}\overset{N}{\underset{m=1}{\sum }}e_{t}\left(m\right),e_{t}\left(m\right)=\{\begin{array}{ll} 1 & \mathrm{if}\left|{\mathrm{\beta ̂}}_{m}-\beta_{m}\right|\geq t\\ 0 & \mathrm{otherwise} \end{array}$$ 3

where *N* is the number of molecules, *β̂*_*m*_ is the prediction of the model, and β_*m*_ is the ground truth value of the molecule *m*. The threshold of error has been chosen to be *t* = ln2. This value is the smallest interval between two measured concentration levels in the experimental data set. Hence, the score *A* measures the percentage of correctly predicted β_*m*_ in our experiment. We also use this score for the *logIE* data set, replacing β_*m*_ with the *logIE* value.

### MLP, Decision Tree, And Random Forest

We train the first family of ML models (Multi-Layer Perceptron, Decision Tree, and Random Forest) using all molecular descriptors provided by the open-source library ”Padel”.^14^ The results of the regression for all models are presented in Table 1. Among the 1,444 features available, some of them are not informative for predicting β. This can confuse neural networks that can find spurious correlations, even more with the small data set size (191 molecules). It is particularly visible for the MLP. This model has far more parameters than data points and cannot provide a robust prediction from the possible many irrelevant or highly correlated features. While it has an accuracy of 56% for LOOCV and 51% for 5-fold CV, its MAE and MSE score explode due to predictions on a few molecules that are order of magnitude far from the true value. Random Forests and Decision Trees are more robust to small sample sizes with many features because they perform implicit feature selection and do not rely on gradient-based optimization like MLPs. In any case, reducing the high dimensionality of the input can be beneficial to ML models.

**Table 1:** Prediction Results of the Non-Graph-Based ML Methods for Predicting *β* ^a^

Evaluation LOOCV	MLP	Decision Tree	Random Forest
Regression on all features			
Accuracy, %	56 ± 1.3	59 ± 0.8	63 ± 0.6
MAE	32.081 ± 4.027	0.793 ± 0.014	**0.710** ± **0.003**
MSE	159,440 ± 43,935	1.309 ± 0.036	1.045 ± 0.006
Regression on the main features			
**100%**			
Accuracy, %	56 ± 1.6	55 ± 0.3	65 ± 0.8
MAE	0.926 ± 0.018	0.868 ± 0.004	**0.700** ± **0.003**
MSE	2.495 ± 0.344	1.432 ± 0.012	1.035 ± 0.006
**75%**			
Accuracy, %	53 ± 3.0	53 ± 4.6	62 ± 1.7
MAE	0.996 ± 0.090	0.933 ± 0.071	0.741 ± 0.032
MSE	2.407 ± 0.653	1.875 ± 0.396	1.151 ± 0.124
**50%**			
Accuracy, %	48 ± 6.1	52 ± 6.8	58 ± 3.5
MAE	1.067 ± 0.190	0.989 ± 0.117	0.758 ± 0.054
MSE	3.114 ± 1.616	2.057 ± 0.379	1.131 ± 0.169
**25%**			
Accuracy, %	38 ± 9.3	56 ± 7.0	57 ± 5.6
MAE	1.197 ± 0.294	1.005 ± 0.236	0.779 ± 0.068
MSE	4.125 ± 5.222	2.150 ± 0.983	1.075 ± 0.243

Evaluation 5-fold CV	MLP	Decision Tree	Random Forest
Regression on all features			
Accuracy, %	51 ± 8.2	60 ± 3.5	60 ± 7.2
MAE	33.393 ± 61.339	0.854 ± 0.148	0.738 ± 0.102
MSE	168,458 ± 336,031	1.533 ± 0.634	1.090 ± 0.515
Regression on the main features			
Accuracy, %	48 ± 7.4	58 ± 6.4	62 ± 4.9
MAE	0.982 ± 0.149	0.878 ± 0.100	0.723 ± 0.136
MSE	2.289 ± 1.185	1.635 ± 0.524	1.068 ± 0.554

a LOOCV: Leave-One-Out Cross-Validation, MLP: multi-layer perceptron. The results are reported for both LOOCV and 5-fold Cross-Validation. Two types of results are shown, one with training on all the 1444 molecular features “all features” and the second with a reduced set of 11 most important features (according to Random Forest), “the main features”. In addition, we performed experiments on different training set size, from 25% to 100% for LOOCV with 11 features. Results with the highest accuracy and lowest mean absolute error (MAE) and mean square error (MSE) are written in bold. Random Forest trained with LOOCV and 11 main features is the best overall model and the most stable. *Remark:* The extremely high MAE for the MLP is due to a few molecules (2–3, depending on the run) with completely wrong predictions (several orders of magnitude).

We use our Random Forest model to estimate the most important features (as stated in the previous section) in order to reduce the input size and increase its quality. The evaluation revealed more than 500 features have no influence on values of β (37% of features). The main feature identified by the model is ”SpMAD_Dzs”, the Barysz vertex-distance matrix.^21^ This matrix includes aggregated information about the molecular structure. For more information on the most important features, see Figures SI1 and SI2 in the Supporting Information. After evaluating the importance of features, we retrained the Random Forest model using only the main features. We attempted several configurations and found that 11 features provide a good compromise between accuracy and number of features. No further improvement of the results was achieved beyond these 11 features (Figure SI3, Supporting Information). Random Forest has consistently better performance for the prediction of β, followed by Decision Tree and then MLP. The best results are obtained using the reduced number of 11 main features, identified by the Random Forest model. The results are robust across all training approaches, with low variances and similar results for the different types of trainings. We note a decrease of the results as the data set becomes smaller, which is expected for any ML method. Consequently, we expect better performance as the data set grows.

### Graph Neural Network

The main advantage of the GNN is that it can create its own representation of the molecular structure. It learns to focus on meaningful structural properties for the task at hand. Moreover, we do not need to provide a list of low- and high-level features such as the ones obtained from RD-KIT. A standard approach to preprocess molecule data for GNNs is to create a graph where nodes are atoms and edges represent atomic bonds. Elemental properties (atomic descriptors) within each molecule are then added to each node as a feature vector per node. However, some information about the overall molecule can be beneficial for the GNN (see Lutchyn et al.^11^); in our approach, we follow this suggestion and combine atomic descriptors with four molecular descriptors. Testing on open data sets,^11^ shows that this combination provides better results than having only molecular descriptors in nongraph approaches or only atomic level information on the nodes for GNN models.

The results of the GNNs for predicting β are summarized in Table 2. First, average pooling is consistently better, showing no advantage of using a single node over average pooling for our data set. We can assume that β does not depend on a particular substructure of the molecule, at least not one that is encoded at the beginning or end of the SMILES string. Second, the results for our GNN are much better than the nongraph ML approaches, with lower MAE and excellent accuracy, approximately 40% reduction in MAE compared to Random Forest. This is also shown in Figure 3: The error range is narrower for the GNN, as indicated by the lower MAE score reported in the table. The GNN performs better and has a small error even for molecules for which Random Forest is unable to provide a good prediction. We also note two outliers with large deviation for which the GNN fails to give a reasonable prediction. Finally, we visualize the error with respect to the value of β, to see whether a better prediction can be achieved for a particular range of values. As shown in Figure 4, the GNN is more accurate than Random Forest, and it maintains good accuracy over the whole range of values, especially for small values of β. Random Forest fails to correctly predict small βs (overestimation), and to some extent, large ones too (underestimation). This observation aligns with the results from Tables 1 and 2, which show that the levels of accuracy for Random Forest and the GNN (average pooling) are 62% and 79% by 5-fold cross-validation, respectively, whereas for the same models the MSE is around three times higher for Random Forest.

**Figure 3:**
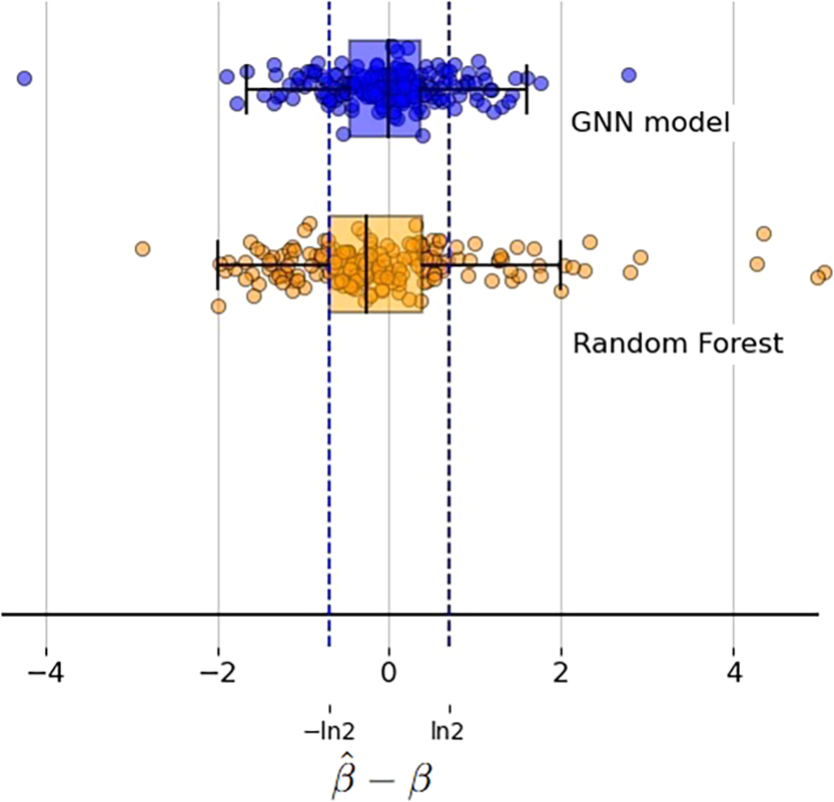
Box plot showing the deviation in the error (*β̂* – β) between the prediction *β̂* and the true value of β for the GNN and the best nongraph ML model: Random Forest. Each dot is the error for a sample, with results shown from the 5-fold CV.

**Table 2:** Scores for the Prediction of *β* with the GNN under Different Configurations: Selecting Single Nodes or Average Pooling^a^

5-fold CV			
	MAE	MSE	Accuracy, %
1^*st*^ node	0.569 ± 0.067	0.625 ± 0.179	71 ± 5.6
2^*nd*^ node	0.496 ± 0.042	0.442 ± 0.087	76 ± 4.9
Last node	0.568 ± 0.078	0.604 ± 0.167	67 ± 8.3
Average pooling	0.439 ± 0.072	0.316 ± 0.083	79 ± 5.4

LOOCV			
	MAE	MSE	Accuracy, %
1^*st*^ node	0.488 ± 0.019	0.502 ± 0.036	77 ± 2.8
2^*nd*^ node	0.395 ± 0.015	0.306 ± 0.019	83 ± 3.0
Last node	0.498 ± 0.029	0.505 ± 0.053	74 ± 2.3
Average pooling	0.362 ± 0.011	0.241 ± 0.009	85 ± 2.4

a For the first, second, and last nodes, the node corresponds to the first, second, and last atoms in the SMILES encoding chain respectively. These results are obtained using 5-fold CV and LOOCV. The deviation is computed over five experiments (five trained models).

**Figure 4:**
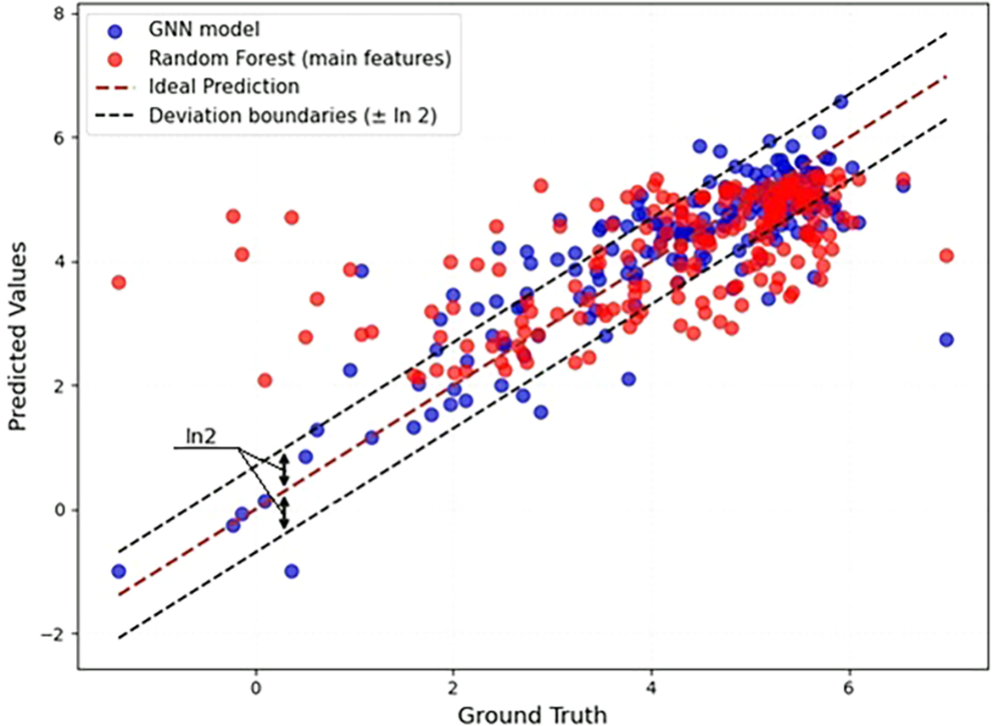
Ground truth vs predicted β for the GNN model (blue) and Random Forest (red). The solid line depicts the correct prediction of β, and dashed lines correspond to the deviation limit for our accuracy measure. Most of the predictions of the GNN fall into the ±ln 2 range and have smaller deviation from the ideal prediction than those of Random Forest. Results from 5-fold CV.

### Evaluation on the Ionization Efficiency “*logIE*” Data Set

The library containing *logIE* values is described in.^10^ In this data set, we compare the model from,^10^ which we call the “SOTA” model, to the best performing nongraph model (Random Forest Regression) and our GNN.

We show the results of the three models in Figure 5. Overall, the best model is the GNN. This is confirmed by the MAE score, as shown in Table 3. A striking result, visible in Figure 5 is the presence of two outliers in the GNN predictions. All of the predictions are better than those of the other models, with the exception of two molecules. These molecules are tylosin and flunitrazepam. To isolate their influence, we also introduce an MAE* score, which is just the MAE without these two outliers. Figure 6 shows the predictions for the different values of *logIE*. All three models appear to struggle to predict the *logIE* with negative values well. This may be due to the extremely small number of samples in this range. For positive values of *logIE*, the error is homogeneous and there is no particular range where it performs better or worse. This is true for the three models.

**Figure 5:**
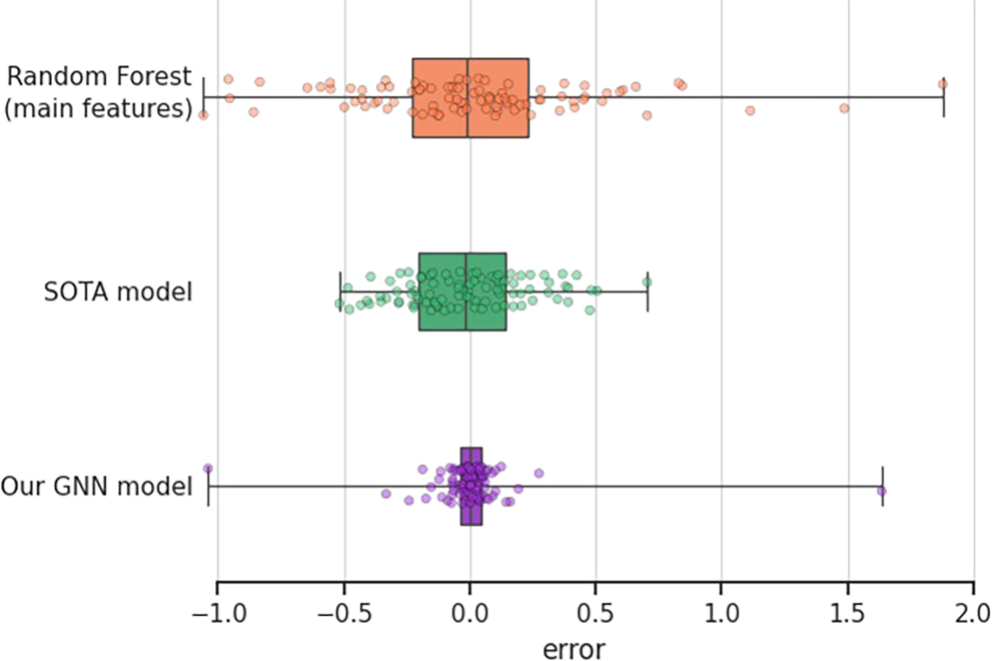
Prediction errors for the *logIE* library for the three test models. The GNN model has the best prediction. The two extremely wrong predictions by the GNN are the molecules tylosin and flunitrazepam.

**Figure 6:**
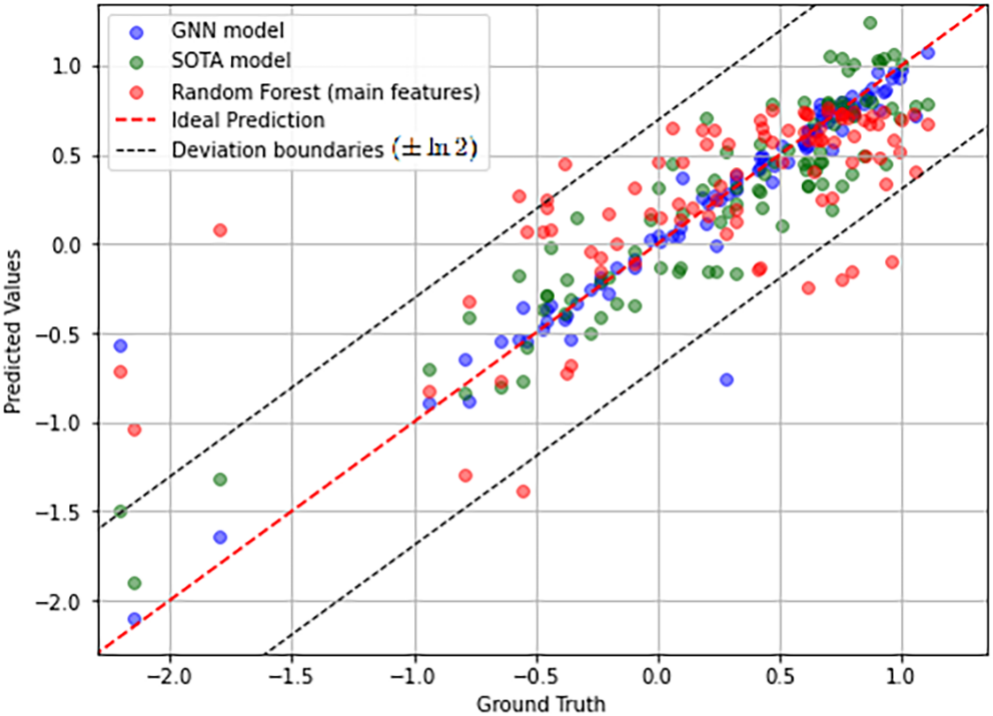
IE prediction result (LOOCV) using our GNN model (blue) and Random Forest for the main features (red) compared with the SOTA model (green).

**Table 3:** Results for the Prediction of *logIE* for the Different Models^a^

Model	MAE
GNN 1^*st*^ node	0.123 ± 0.026
GNN 2^*nd*^ node	0.120 ± 0.026
GNN last node	0.108 ± 0.024
GNN avg. pooling	0.108 ± 0.025
RF (all features)	0.145 ± 0.014
RF (main features)	0.211 ± 0.018
SOTA^10^ (LOOCV)	0.197

Model	MAE*
GNN 1^*st*^ node	0.096 ± 0.017
GNN 2^*nd*^ node	0.090 ± 0.014
GNN last node	0.089 ± 0.011
GNN avg. pooling	0.086 ± 0.013
RF (all features)	0.145 ± 0.015
RF (main features)	0.212 ± 0.018
SOTA^10^ (LOOCV)	0.190

a The deviation is computed over five experiments (five trained models). The MAE* score is the MAE score without the two outliers: tylosin and flunitrazepam. The GNN model outperforms the other models.

## Discussion

We have developed ML models that can predict the concentration level of drugs confidently identified in whole blood. The models were trained using a data set of 191 drugs spiked into whole blood, which was originally acquired to determine the decision points of these drugs for a specific forensic drug screening method. The data set is consequently suitable to be reapplied here to develop a GNN model to estimate concentration level in whole blood measurements from the same screening method. ML models perform best with very large data sets for training, which might be why they have not been used for concentration predictions, for which the data sets are costly to perform. As our results show, the models have outliers and would undoubtedly benefit from a larger number of drugs for training. Increasing the data set would improve generalization and limit overfitting. Prospective validation using authentic forensic casework data would provide additional evidence of robustness under real-world conditions and heterogeneous case scenarios. In practice, however, the use of authentic case data for machine-learning validation in a research context is associated with substantial administrative and procedural obligations under data protection and other legislation.

Of the nongraph-based ML models tested here, Random Forest performed best, although Decision Tree provides very close results in the 5-fold CV case and might be better on some subsets. A Random Forest model was previously found to be the best-performing approach in a recent interlaboratory study comparing different methods for quantification without using reference standards.^9^ However, in the current study, the GNN was superior to all tested nongraph models. Despite the small size of the data set, the accuracy of the GNN was high and β_*m*_ could be predicted within ± ln2 for most molecules. This would mean that at a given *s̅*_*m*_, the concentration in whole blood would be predicted within a factor of 2. Thus, a predicted value of 0.10 mg/kg would be in the range of 0.050–0.20 mg/kg. If a rare drug is confidently identified in a forensic case from LC-HRMS drug screening and the concentration is predicted to be 0.10 mg/kg, for example, it would be worth purchasing a reference standard if the toxic level in blood is 0.20 mg/kg - but it would probably not be worth purchasing if the toxic level is 8.0 mg/kg. This is a highly acceptable prediction range considering that toxic concentrations of different drugs can easily cover 5 orders of magnitude.^22^

There are no SOTA models for the quantitation of drugs in whole blood without reference standards, but since IE should be an important parameter in β, we benchmarked our ML models in the *logIE* data set. Here, we saw that the GNN outperformed the current SOTA model for IE prediction. However, it is a limitation that this evaluation was performed only on the subset of molecules in the data set for which we could source SMILES.

The training set is representative of drugs relevant for forensic drug screening but does not cover groups of chemicals such as environmental pollutants or plant toxins, and metabolites are also not well represented. For this reason, the application domain of this model is limited to drug-like molecules that are chromatographically retained and confidently identified to ensure accurate molecular representation. Molecules that are similar to drugs that were used to train the model should give the best predictions, and it is not recommended to use the model for completely novel drug classes. This is illustrated in results from the GNN used for *logIE* prediction. Flunitrazepam from the test set was an outlier in the prediction, but also the only nitrobenzodiazepine in the data set so the model had not seen this type of molecule. Furthermore, molecules that predominantly form adducts other than [M + H]^+^ and/or have pronounced *in-source* fragmentation could give higher prediction error, as noted by Liigand et al.^8^ Since confident identification is a prerequisite, it is implied that there is a certain signal intensity to produce sufficient identification parameters. Tentative identified drugs that are retained chromatographically with good library spectral matches are suitable candidates for semiquantitative estimation using this model. From a toxicological and epidemiological perspective, the investigation of new psychoactive substances is justified irrespective of quantitative estimates, particularly for highly potent compounds such as nitazenes. A further semiquantitative approach of future interest in forensic toxicology is the use of fragmentation data to prioritize unknowns without having to first identify the compounds. This approach has been demonstrated for food-contact materials,^23^ but the requirements for defensibility and throughput in forensic laboratories currently make it impractical. Considering how forensic drug screening is performed today,^2^ our presented GNN is an ML application that can serve as automated decision-support in high-throughput settings.

## Conclusion

This study introduces a novel approach for estimating drug concentrations in whole blood using graph ML. The proposed GNN outperformed other ML models and achieved an accuracy suitable for applications in forensic toxicology. The model was also benchmarked on an openly accessible IE data set, where it outperformed the current state-of-the-art method. These experiments were conducted on a relatively small data set; expanding the training data to include more molecular structures would help validate its broader applicability in toxicology.

The proposed workflow is ready for integration into high-throughput forensic drug screening pipelines to estimate concentration levels of confidently identified compounds. This is particularly valuable for newly emerging or rarely encountered drugs, for which reference materials may be difficult or impossible to obtain. Rapid, informed decisions about whether reference materials are necessary—especially when predicted concentrations fall within therapeutic ranges—can shorten turnaround time by weeks and reduce the substantial analytical workload. Overall, the findings demonstrate the strong potential of ML to provide automated decision support in forensic toxicology.

## Supplementary Material



## Data Availability

The graph neural network model trained in this work is open-source: model available on Github https://github.com/uitml/GNN-for-drug-concentration, inspired by the TchemGNN model https://github.com/uitml/TChemGNN. The data set with molecules and their signal-to-concentration ratioβ is publicly available on Zenodo.^24^

## Notes

Additional experimental details on sample preparation, instrumentation, and the Random Forest models. Additional figures of the main molecular features according to our Random Forest Regression model (Figure SI1); pairwise distributions with KDE (kernel density estimation) plots between Random Forest’s main features ‘SpMAD_Dzs’, ‘ETA_Eta_L’, ‘GATS1v’(Figure SI2); and accuracy versus numbers of main features given as input to Random Forest Regression (Figure SI3) (PDF)
