# Supplementary material for: Graph Machine Learning Can Estimate Drug Concentrations in Whole Blood from Forensic Screening Results
Source: Anal Chem. 2026 Mar 18;98(12):9115–23. doi: 10.1021/acs.analchem.5c07428 (PMC13044884; doi:10.1021/acs.analchem.5c07428)
Supplement: Supplementary file 1 [file ac5c07428_si_001.pdf]

Supporting information for:

Graph machine learning can estimate drug concentrations in whole blood from forensic screening results

Tetiana Lutchyn[1], Marie Mardal[2,3]*, Michael Nedahl[3], and Benjamin Ricaud[1]

[1]Department of Physics and Technology, The Arctic University of Norway, 9019 Tromsø, Norway

[2]Department of Pharmacy, The Arctic University of Norway, 9019 Tromsø, Norway

[3]Department of Forensic Medicine, University of Copenhagen, 2100 Copenhagen, Denmark

*Email: marie.mardal@sund.ku.dk

Address:

Department of Forensic Medicine

University of Copenhagen

Frederik V's vej 11,

2100 København Ø

Denmark

Table of Contents

Materials and methods 3

Samples 3

Instrumentation 3

Random forest most important features 4

Figure SI1: Main molecule features according to our Random Forest Regression model. 4

Figure SI2: The pairwise distributions with KDE (Kernel density estimation) plots between Random Forest's main features 'SpMAD_Dzs', 'ETA_Eta_L', 'GATS1v'. 5

Figure SI3: Accuracy versus numbers of main features given as input to the Random Forest Regression. 6

Materials and methods

Samples

The blank whole blood was tested on various in-house methods prior to analysis for common therapeutic and illicit drugs and found free for drugs not commonly found in blank blood (caffeine, nicotine, some antihistamines)

Dilution rows at seven concentration levels of 13 different standard mixtures were prepared by serial dilution in 1:1 methanol:water. Each standard mixture had 7-35 different drug targets (median: 20) with a total of 231 drug targets. Diluted mixtures were spiked to blank whole blood samples and vortexed. The concentration range was typically 0.001-0.1 mg/Kg.

Spiked whole blood samples were extracted by a previously developed protein precipitation protocol using a Tecan Freedom EVO 200 robotic platform (Tecan Group Ltd, Männedorf, Switzerland) [DOI: 10.1093/jat/bks014]. Briefly, 0.1 g of blood was transferred to a 96-well plate, followed by the addition of ISTD and 700 µL acetonitrile; the plate was then agitated on an orbital shaker, added 50 µL 10 % formic acid in acetonitrile, and centrifuged at 1,000×g. Supernatants were evaporated to dryness under a stream of nitrogen at 35°C, reconstituted using 100 µL of water/methanol/formic acid (74:25:1, v/v/v) and transferred to a secondary 96-well plate for analysis by LC-HRMS.

Instrumentation

Analysis of whole blood samples were performed using an Ultra-High Performance Liquid Chromatography - quadrupole Time-of-Flight - Mass Spectrometry (UHPLC-qTOF-MS) system consisting of an ACQUITY UPLC I-Class coupled to a Xevo G2-S QTOF (Waters, Milford, MA, USA). Analytes were separated using an ACQUITY UPLC® HSS C18 (1.8 µm 2.1 × 150 mm) column (Waters) with a flow rate of 0.4 mL/min at 50°C. The mobile phases consisted of 5 mM aqueous ammonium formate buffer (pH 3) (A) and 0.1% formic acid in acetonitrile (B). The gradient was 13% solvent B (0–0.5 minutes), 13–50% solvent B (0.5–10.0 minutes), 50–95% solvent B (10.0–10.75 minutes), 95% solvent B (10.75–12.25 minutes), 95–13% solvent B (12.25–12.5 minutes) and 13% solvent B (12.5–15 minutes). The injection volume was 3 µL.

The mass spectrometer was operated with a Z-spray in positive electrospray ionization mode ESI^+ using the following source conditions: desolvation gas flow 800 L/h, desolvation temperature of 400°C, cone gas flow 20 L/h, source temperature 150°C, capillary voltage 0.80 kV, cone voltage 25 V and argon as the collision gas. Data were acquired using data-independent acquisition mode with elevated collision energy. The low collision energy was set at 4 eV, and the high collision energy ramped from 10 to 40 eV. The range of mass-to-charge (m/z) was 50–950. Mass calibration was performed weekly with 5 mM sodium formate solution in 2-propanol:water (90:10, v/v). Lock mass correction was performed with leucine enkephalin as reference mass at m/z=556.2766 Da.

Random forest most important features

The 11 most important features selected by our Random Forest model are shown in Fig. SI1. For the 3 most important features, the pairwise distributions with kernel density estimation (KDE) are depicted in Fig. SI2. It shows some light correlation between the features. We trained the Random Forest on an increasing number of main features and show the accuracy scores in Fig. SI3. From the plot, 11 features seem to be the best compromise between the accuracy and number of features.

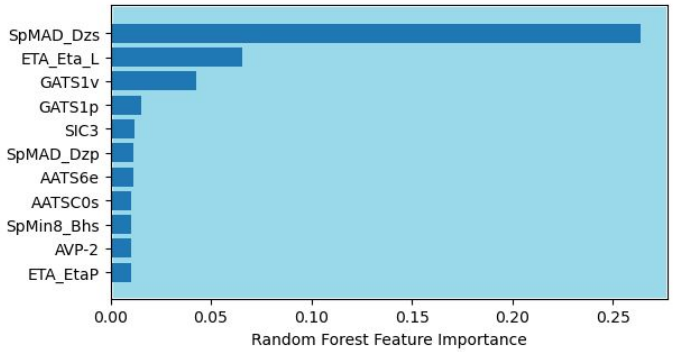

Figure SI1: Main molecule features according to our Random Forest Regression model.

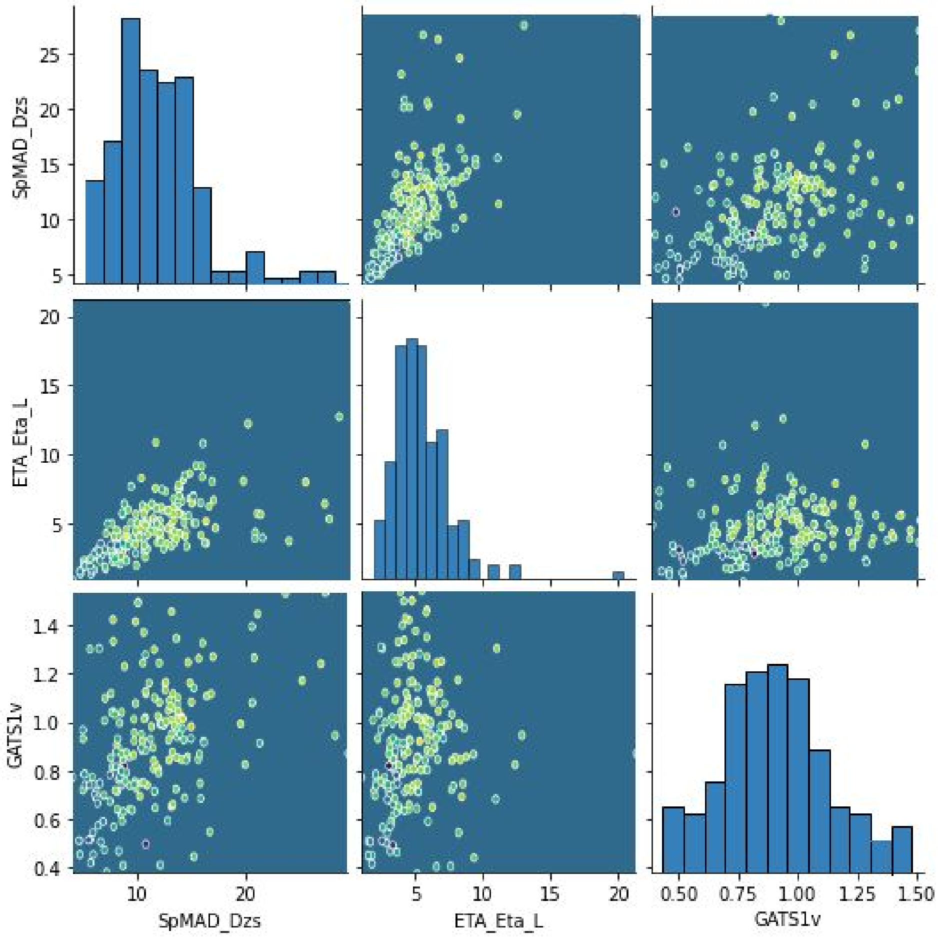

Figure SI2: The pairwise distributions with KDE (Kernel density estimation) plots between Random Forest's main features 'SpMAD_Dzs', 'ETA_Eta_L', 'GATS1v'. Correlations can be seen, showing that they share some common information.

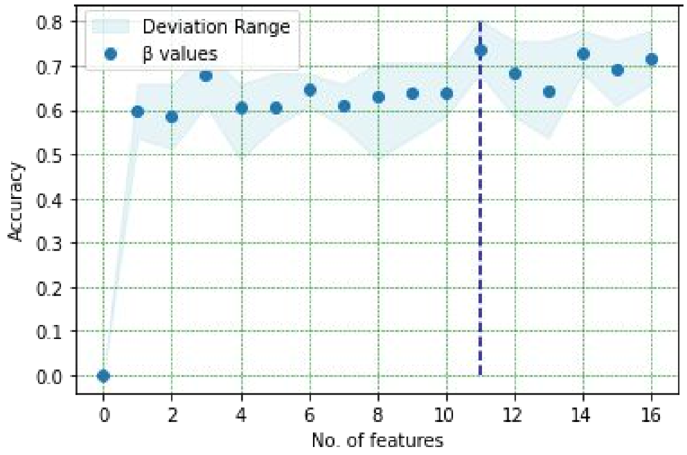

Figure SI3: Accuracy versus numbers of main features given as input to the Random Forest Regression.
